# Ways of promoting health to patients with diabetes and chronic kidney disease from a nursing perspective in Vietnam: A phenomenographic study

**DOI:** 10.3402/qhw.v11.30722

**Published:** 2016-05-10

**Authors:** Lotta Pham, Kristina Ziegert

**Affiliations:** School of health and welfare, Halmstad University, Halmstad, Sweden

**Keywords:** Diabetes and chronic kidney disease, in-patient care, health promotion, physical activity, nursing, phenomenography

## Abstract

Health promotion plays an important role in the management of diabetes and chronic kidney disease, especially when the prevalence of the disease is rising in Vietnam. Nurses have been identified to be the front figure in health promotion; however, little is written about how nurses in Vietnam work with these issues. Therefore, the aim of this study was to describe nurses’ conceptions about how health is promoted, with special focus on physically activity, for patients with type 2 diabetes (T2DM) and/or end-stage renal disease (ESRD). Individual interviews were done with 25 nurses working at two major hospitals in Hanoi, Vietnam. A phenomenographic approach was used to analyse the interviews. Nurses described how creating positive relationships and supporting patients to take part in their social context promoted health. Health was also promoted by educating patients and relatives about health and disease and by supporting patients to be physically active. The findings indicate that the Vietnamese nursing knowledge about health promotion needs to be gathered, and health promotion needs to be further integrated in the education. Further research is necessary to examine patients’ knowledge and attitudes about health and the efficiency of various health-promoting strategies in the Vietnamese context.

Health promotion is a central part of nursing (Piper, 2009) and the nursing profession has been identified to have the potential to be the frontline in the expansion of health promotion (Whitehead, 2009). At the same time, health promotion is a contested and well-debated concept (Piper, 2009).

Many nurses think of health promotion as a part of the nursing role, but health promotion is often viewed as health education (Kelley & Abraham, 2007). Health education is described as a planned process with the aim to attain health- and illness-related learning, and as an important part of an empowerment model of health promotion. Health promotion is defined as processes that facilitate people to enhance and improve control over their health (World Health Organization [WHO], 1986), and includes both health education and strategies to meet social and environmental determinants of health. Environmental factors are affecting health both directly, such as exposure for toxic materials and shortage of food, or indirectly, such as lack of facilities for exercise, therefore leading to lower levels of physical activity (Green & Tones, 2010). Supportive environments are an obvious concept in health promotion, and are described to make the physical, social, economic, and political environment supportive to health (WHO, 1991).

## Nursing and healthcare in Vietnam

The Vietnamese culture and religious beliefs, which are influenced by Buddhism, Taoism, and Confucianism, is described to affect nursing and healthcare in Vietnam. Family members have a great role in Vietnamese healthcare and primarily provide traditional treatments at home. Even at the hospital, the family has a major part in the care and is responsible for tasks such as nutrition, bathing and bedding (Pron, Zygmont, Bender, & Black, 2008).

The many years of war, when nursing practice was frequently performed by people with basic first aid knowledge, characterizes the nursing practice in Vietnam (Jones, O'Toole, Nguyen, Tran, & Pham, 2000). In the clinical setting, nursing interventions are focused on medical care and activities associated with the implementation of doctors orders and medical treatment (Pron et al., 2008). The nursing education varies over the country, both in content and length (Hill & Crow, 2013). A 4-year bachelor nursing degree is provided by 14 universities and approximately twice as many junior colleges provide 3-year training for nurses (Van der Velden, Van, Quoc, Van, & Baron, 2010).

## Type 2 diabetes and end-stage renal disease in Vietnam

In Vietnam the prevalence of diabetes mellitus (DM) is rising and is now estimated to be about 6% (Pham & Eggleston, 2015), compared to the prevalence of DM in the world which is 1.9% (Pranoto, 2014). The rising prevalence of diabetes may be explained of an aging population, urbanization, increased incidence of obesity and physical inactivity (Pham & Eggleston, 2015). Diabetes is a major cause of chronic kidney disease (CKD), and approximately 6% of the people in Vietnam suffer from CKD (Van, Duangpaeng, Deenan, & Bonner, 2012). Early stages of CKD often give little or no symptoms and, therefore, CKD often leads to end-stage renal disease (ESRD). Every year, 8000 new patients in Vietnam reach ESRD where haemodialysis or peritoneal dialysis is the most common treatment (Van et al., 2012).

The Vietnamese society has changed rapidly, with an economic growth and poverty reduction, and in 2010, the country attained lower middle-income country status (WHO, 2012). Nevertheless, Vietnam has still to tackle inequalities in health related to poverty (Baumann, Blobner, Ta, & Pham, 2006; Gien et al., 2007; Ito et al., 2008). Poverty is a barrier in the treatment of patients with diabetes, which leads to some patients not following the required treatment (Baumann et al., 2006). Poverty is also a barrier in the management of ESRD. Haemodialysis is an expensive treatment and this combined with low health insurance coverage leads to the fact that many patients without insurance cannot continue the treatment, and die after some weeks (Ito et al., 2008).

## Physical activity in the promotion of health

Promoting physical activity is an important way to promote health as physical activity has benefits for individuals’ health in all ages (Kohl et al., 2012). In Vietnam many adults are physically inactive and not achieving the minimum recommendation of doing 30 min moderate-intensity physical activity for at least 5 days a week. Participation in leisure time physical activity is also limited; instead the levels of physical activity are linked to transport and occupational activities (Trinh, Nguyen, Dibley, Phongsavan, & Bauman, 2008).

Physical inactivity has been described as a contribution factor of the rising prevalence of diabetes in Vietnam (Pham & Eggleston, 2015). Physical inactivity is also described as a risk factor for increased morbidity as elderly with T2DM and a sedentary lifestyle has an increased risk of cardiovascular events (Iijiama et al., 2012). Furthermore, physical inactivity has a negative impact on the quality of life for patients with T2DM (Chyun et al., 2006) but general health and health-related quality of life for patients with T2DM can be improved by regular exercise (Eckert, 2012; Ng, Tai, Goh, & Wee, 2011). Patients with ESRD also have positive benefits of physical activity as physical capacity, psychological status and quality of life can be improved through regular exercise training during haemodialysis (Ouzouni, Kouidi, Grekas, & Deligiannis, 2009). Leg cycling during haemodialysis is an intervention with positive effects on patients’ health with improvements on muscle strength, power, fatigability and physical function (Storer, Casaburi, Sawelson, & Kopple, 2005). Support and motivation by nurses are important factors for patients with ESRD to continue with physical activity (Tobita, Suzuki, Kobayashi, Shimizu, & Umeshita, 2009).

Health promotion, and especially promotion of physical activity, are important interventions, with low costs in the management of both T2DM (Eckert, 2012; Ng et al., 2011) and ESRD (Ouzouni et al., 2009; Storer et al., 2005), diseases where the prevalence is on the increase in Vietnam (Baumann et al., 2006). Internationally, nurses are described as the primary providers of health promotion (Whitehead, 2009). Despite this, almost nothing is known about Vietnamese nurses’ conceptions of health promotion and the promotion of physical activity, and how they work with these issues. Therefore, the aim with this study was to describe the nurses’ conceptions about how health is promoted, with special focus on physical activity, for patients with TDM2 and/or ESRD.

## Method

A descriptive qualitative design with a phenomenographic approach was used in the study. In phenomenography, the focus of research is variation in the way of experience a phenomenon. Variation is present in certain conceptions of a phenomenon due to different ways of seeing, and these conceptions are illuminated by the authors (Marton, 1999). In pheomenography, the participants’ thoughts about a given phenomenon are more important than their number. The phenomenographic method allows conceptions of a phenomenon contained in collected data to be explored by means of analysis and interpretation and sorted into descriptive categories based on referential and structural meanings (Marton 1981; Marton; 1999).

### Participants

The first author was a Swedish registered nurse. A purposeful sample was used to find participants for the study. The study was performed at two national hospitals in Hanoi, Vietnam. In the first hospital specialized in geriatrics and patients with diabetes, the study was performed in the endocrinology department. In the other hospital, the study was performed at the nephrology- and urology department. Two physicians, employed in each hospital, and tutors at the nursing school at Hanoi Medical University, supported the first-author to coordinate the interviews. The two physicians recruited three nursing students and one medical staff, to interpret between English and Vietnamese during the interviews.

Participants were selected by purposive sampling, with differences in gender, education and years of nursing experience. Inclusion criteria included nurses educated and active in Vietnam; exclusion criteria included nurses educated in other countries. The participants were recruited continuously during the study, and all nurses that were asked chose to participate in the study. An overview of the participants is presented in Table I.

**Table I:** Overview of participants.

Sex	Female	23
	Male	2
Age	18–30	18
	31–55	7
Education	2–3 years	21
	4 years or more (bachelor degree)	4
Work place	Endocrinology unit	10
	Nephrology unit	15
	Inpatients	18
	Outpatients	7
Work experience	4 months–1 year	4
	2–5 years	12
	6–14 years	4
	15–20 years	5

### Data collection

Data collection was conducted during five weeks in March and April, 2014. To learn more about healthcare in Vietnam, the author spent some days at each hospital to observe the daily work, both before and during the study process. Data collection was conducted with individual interviews. The interviews were done in English with the help of interpreters who translated the interviews. It was not possible that only one person interpreted all the interviews, due to the interviews being time consuming. Therefore, the four interpreters shifted, and each of them interpreted five to seven interviewers. All interpreters in the present study had Vietnamese as their mother tongue, but with good knowledge of the English language. Their knowledge about the Vietnamese healthcare system and knowledge of medical language in both Vietnamese and English were important in the interpretation of the interviews. This was a sensible part of the study due to the risk of misunderstanding. To improve misunderstanding, a close cooperation with the three nursing students was established. The author and the nursing students met and discussed the data collection and ways to translate the questions before data collection began. The three nursing students followed the planning of the study and had opportunities to share opinions regarding the research question and the interview guide. This was important to make the study viable in the Vietnamese context. The medical student was introduced later, due to time limitations for the nursing students, and had the opportunity to read the project plan and the interview guide before the data collection began.

The interviews were held at the hospitals where the participants worked. The hospitals were considered to be the most suitable place to perform the interviews, being the best place to coordinate the participants and the interpreters’ schedules.

The length of the interviews ranged from 15 to 40 min. The interviews were recorded with a smartphone and the author took notes.

Sjöström and Dahlgren (2002) described semi-structured interviews as the preferred method to collect data in a phenomenographic approach. In the present study, the interviews were semi-structured and were done with the help of an interview guide. The interview guide had two themes, health promotion and physical activity, with three main questions each (Table II). Examples include “Can you explain …,” “What do you mean …,” “How do you think …,” etc. Three pilot interviews were performed to test the interview guide. The pilot interviews went well and no modifications were carried out in the interview guide. Therefore, these interviews were included in the study.

**Table II:** Six open questions used in the interview of the 25 informants.

1.	What does health promotion means to you?
2.	What do you think about the benefits of physical activity for patients with diabetes and/or end-stage renal disease?
3.	What do you think about health promotion as a part of nursing?
4.	What methods do you use or think you can use to promote physical activity to patients with diabetes and/or end-stage renal disease?
5.	What do you think about your possibilities to promote health to your patients?
6.	What kind of difficulties do you face in work when motivating patients to be more physically active?

### Data analysis

After the interviews were performed, the author listened to the interviews again, and the translated answers were transcribed verbatim into English. This was a long process where interviews were listened through several times to achieve the correct transcription. To validate the verbal translation, some interviews were transcribed verbatim in Vietnamese and translated to English by one of the interpreters who had not interpreted the current interview. This was important to catch any conceptions that may have been missed in the verbal translation. The data analysis was done with the transcribed material. The co-author was well versed in the research approach and acted as a co-analyser during the categorization. The analysis of the data followed Dahlgren and Fallsberg's (1991) recommendations:

1. *Familiarization*. To become familiar with the data, the transcripts were read through several times.
2. *Condensation*. In this step, the condensation was performed, where longer statements were reduced to find the core of each dialogue. Statements that answered the aim of the study were identified and collected in a table on the computer.
3. *Comparison*. The statements were compared to find similarities and variations in the conceptions of the core aspects of health promotion, and the nurses’ role in promoting physical activity.
4. *Grouping*. Similar answers were grouped together to find out preliminary conceptions which answered the aim of the study.
5. *Articulating*. Here conceptions were compared and grouped into four descriptive categories covering nurses’ conceptions of how health was promoted for patients with T2DM and/or ESRD.
6. *Labelling*. In this step, the four descriptive categories were named.
7. *Contrasting*. This was the final step where a contrastive comparison of the categories was performed to find the unique characteristics of each category.

### Ethical considerations

The study was approved by the ethical committee at Halmstad University in Sweden, Dnr: 012014192. Polit and Beck (2012) describe three ethical principles to protect the participants in a study: beneficence, respect for human dignity, and justice. Beneficence implies that participating in the study will not lead to any harm for the participants. The study design in the present study was not considered to cause any harm for the participants. Possible benefits from the study include the opportunity to share conceptions and reflect about the daily work.

The principle, respect for human dignity, comprises the right to self-determination and the right to full disclosure (Polit & Beck, 2012). In the present study, self-determination was attained by the fact that participating was voluntary; participants were informed about the study both written and orally in Vietnamese, and had the possibility to ask questions about the study and withdraw from the study at any time. A written consent to participate was given. Polit and Beck (2012) describe that an informed consent is important and includes that the participants have been informed, that they have understood the information about the research, and that they have had the opportunity to either participate or not. Full disclosure was attained by the information given in the information letter, and the verbal information before each interview.

Justice, as an ethical principle in research, deals with issues like the right to fair treatment and the right to privacy (Polit & Beck, 2012). In the present study, a purposeful sample was used and only nurses working with T2DM and/or ESRD patients were selected to participate. The selection of the participants was done to catch variations in the conceptions of the research question. Right to privacy was attained by the fact that all material from the interviews was handled confidentially, and all material was kept in a locked cabinet at Halmstad University. Only the researcher and the interpreter knew about the identity of the participants. During the processing of the material, all interviews were coded with participant 1, 2, 3, etc.

## Findings

The nurses described health promotion as an important part of nursing and that nurses play an important role in health promotion for patients with T2DM and/or ESRD. The nurses had different conceptions about how health was promoted. Some nurses meant that it was very important for all patients, while some meant it depended on the patients’ current health status. Health promotion was described as a fairly new concept for nurses in Vietnam. Some nurses thought that it was difficult to express their conceptions about the health promotion concept and some nurses expressed a wish to learn more about health promotion to improve their skills in this area.

The findings formed four descriptive categories: health was promoted by creating a positive relationship, health was promoted by supporting patients to take part in their social context, health was promoted by educating patients and relatives, and health was promoted by supporting patients to be physically active.

### Health was promoted by creating a positive relationship

This conception describes how a positive relationship was created between a nurse and a patient with T2DM and/or ESRD, and constituted a resource to promote health. The nurses described that they were closer to the patients than the physicians, as they were more available at the hospital and spent more time together with the patients. Therefore, the nurse had the possibilities to promote health through this close relationship. The nurses described promoting health as an approach at the patient meetings, to connecting to the patients and facilitating the communication. The nurses expressed the relationship between the nurse and the patient as positive; to be positive and use humour was a way to connect with the patients.

I can do my work with the best energy and to be positive. It will be more effective. Talk with the patient and have time with the patient about what they want. And when I talk with the patient, the funny will help the patient relax. (Participant 7)

The nurses described various difficulties in creating a positive relationship with the patients. The patients’ physical or mental health conditions, such as severely ill patients, or patients with depression or Alzheimer disease, were sometimes a barrier in creating this positive relationship. Some nurses described difficulties in connecting to patients from ethnic minorities; due to the language barrier and cultural differences. The nurses also described patients’ lack of motivation to listen to the nurses’ advice and adapt to a healthy lifestyle as a barrier to promote health through a positive relationship.

The nurse cannot connect to the patient because they do not want to, they do not need anything to promote their health. They maybe have a depression. Difficulties that exist are that many patients do not want to listen to advises. (Participant 1)

Patients are too old, have severe diseases, patients think that they are coming to the hospital to have treatment. (Participant 17)

### Health was promoted by supporting patients to take part in their social context

This conception deals with the nurses’ ambitions to support patients with T2DM and/or ESRD to take part in their social context. Nurses described that they could promote health by supporting patients to come closer to the community and have them easier to talk with other patients. The nurses also described how they could promote health by supporting patients to take part in various activities, patient clubs or use media; both in and outside the hospital.

Health promotion means that the nurse helps the patients to take part in some activities. And if some organizations organize some campaigns and big activities, the nurse will advise the patient to join these campaigns. It can be the hospital or some organizations that arrange the campaign or activities for the patients. (Participant 20)

There are many clubs for the patient, clubs for diabetes patients, and clubs for hypertension patients. Sometimes, a nurse or a doctor can come and talk to the members. I also think media has an important role, like television or posters. (Participant 10)

The nurses described how they handled the collectivistic society where support from others plays an important role in the health-promoting work. Support from other patients as well as support from relatives was mentioned to be important to promote health for the patients. Relatives and other patients were both described as a resource, but also as a barrier in the nurses’ health-promoting work.

Also give good examples about other patients who have had good benefits, if the patients see a real person with benefits they have increased motivation. Give information to people close to the patient, such as relatives, so they can help with motivating the patient. (Participant 5)

In this hospital, most of the patients are elderly so I must talk to their relatives. Because maybe the patient has Alzheimer disease, so they need help from their relatives. Some people care about their parents, so they listen to the nurses’ advices but some doesn't. (Participant 9)

It was not only the relatives and the family that could be a barrier in the nurses’ health-promoting work. The nurses also described how the patients’ economic conditions sometimes impeded the nurses when promoting health.

Some patients they are so poor and they do not have enough money to buy medicines. (Participant 18)

### Health was promoted by educating patients and relatives

This conception describes how the nurses emphasize that health was promoted through educating patients with T2DM and/or ESRD and relatives about health, disease, and self-care. The nurses considered that patient education was important because more knowledge would lead to a better quality of life as well as better health for the patients. To educate patients about health, disease, and self-care was described as a responsibility in nursing.

Health promotion plays an important role to nurses because if the nurse do not teach or encourage the patient to do physical activity and have a healthy diet, the patient will easier get some complications. And if the patient got pressure ulcer or other complications, and a long stay in hospital, the nurse is responsible for these complications. To teach and educate the patient about a good lifestyle is a responsibility of nursing. (Participant 23)

The nurses provided patient education by answering questions, instructing, and giving advice about health and disease for patients with T2DM and/or ESRD. Nurses described how they could use their voices to encourage healthy behaviour as well as how to take their medicine. The nurses emphasized that patient compliance to the treatment was important and described that their role was to help patients to follow the doctors’ advice. Since relatives had an important role in Vietnamese healthcare, nurses also had to educate them about how to take care of their relative with T2DM and/or ESRD. The nurses emphasized that individual patient education was important.

With ESRD, the disease is individual. I try to teach the patient individually, I focus on the individual. (Participant 16)

Although many nurses emphasized the importance of individual patient education, patient education was often provided in a group with many patients. Both in the endocrinology and the nephrology inpatients unit nurses described that the meetings were often organized once a week. At these meetings, the nurses could educate a large group of patients at the same time; often with all patients at the unit that had the possibility to participate.

Nurses are overworked and too busy. Two nurses take care of over 40 patients, so we cannot focus on the individual. When we educate, we explain for the whole group in one time. (Participant 25)

The nurses also described different ways to provide patient education to the patients with T2DM and/or ESRD and their relatives, such as papers, books, pictures and training classes. Some nurses expressed a willingness to find new ways to provide patient education and suggested technics, such as internet, to connect with patients and their relatives, in their homes.

At the same time, the nurses described that the knowledge of the nurse was not always enough to provide patient education. The nurses requested more knowledge about health, disease, and self-care, as well as nursing methods to promote health. Nurses also described difficulties in educating the patients due to the low social status of the nurse.

Also about the nurses’ knowledge, sometimes the nurse does not have enough knowledge to give advice and educate the patients. (Participant 13)

Maybe it is different in Vietnam, because the patient wants to hear the information from the doctor. If the nurse gives them some advice they may need a doctor to confirm it. (Participant 8)

### Health was promoted by supporting patients to be physically active

This conception deals with the nurses’ conceptions about how health was promoted by supporting patients with T2DM and/or ESRD to be physically active. Health promotion was often described as a way to promote a healthy lifestyle. The nurses’ conceptions regarding physical activity as a way to promote health was further examined, and the nurses described how physical activity was important for patients with both T2DM and ESRD. The nurses described how physical activity improved both physical and mental health as well as preventing complications of the disease.

First about physical health, most ESRD patients have generalized edema. By doing physical activity like walking, it helps to reduce or avoid edema. For patients who lie a long time in bed they have a high risk of pressure ulcer. This will be reduced with physical activity. Physical activity will help to reduce the time in hospital. (Participant 22)

If the patients do physical activity they can relax and have more interest in their lives. (Participant 3)

The nurses supported bedridden patients to be physically active in their beds. To help bedridden patients to move in their bed was described as a nursing intervention aimed to improve health and avoid complications such as decubitus. The nurses also described massage and slight movements as interventions to promote health for severely ill patients.

For patients who lie a long time in bed I can do massage for the patient. I can also help them to do some light movements in their bed, move legs and arms. (Participant 16)

The nurses described barriers in the healthcare setting when promoting health by physical activity in patients with T2DM and/or ESRD. There was a lack of space for patients to be physically active in the hospital area, with not enough areas for walking, jogging, or running. There was also a lack of support devices, such as wheelchairs, crutches, and lifting devices. The nurses also requested training equipment for the patients at the hospital. Despite these barriers in the healthcare setting, the nurses also expressed hope for the future. Many nurses had ideas about how they could work to promote health by physical activity in patients with T2DM and/or ESRD in the future.

In the future, if we have enough facilities, I want to organize some sport activities for the patients, and some competitions for every patient. Then the nurses can encourage patients to take part in these activities. And we can have some small gifts for the patients; these gifts will help the patients to have the power to take part in these competitions. I hope, in the future, the hospital will arrange some activities every month so all the patients, not only in this department, they can come and join these activities, so the patients can extend their relationships with other patients from different departments. It would not be only for this department but the whole hospital. (Participant 20)

## Discussion

The main findings were how the nurses perceived health promoting for patients with T2DM and/or ESRD through a positive relationship, and how the nurses considered that the patients’ social context was important in the health promotion work. The nurses emphasized that health-promoting interventions created both positive relations with patients and relatives, and supported patients to be active and take part in society. This can be linked to how Piper (2009) describes the nurse as an empowerment facilitator, which includes creating a nurse–patient partnership; to help patients cope with the reality and think positively (Piper, 2009). The nurses in Vietnam, therefore, can be seen to promote health by empowering patients with T2DM and/or ESRD to handle their situations and take part in the resources available in the community.

The nurses described that they were close to the patients with T2DM and/or ESRD and how this close positive relationship was a resource in promoting health. This view is consistent with Pender's (2011) description about how nurses constitute a part of the patients’ interpersonal environment and, therefore, is able to decrease or increase health-promoting behaviour (Pender, 2011). Furthermore, according to Lyngå, Fridlund, Langius-Eklöf, and Bohm (2013), collaboration between patients and clinic are proved helpful to patients who described that the patient education helped them understand the relationship between increased health outcomes and other symptoms. This indicates how the nurses are important actors in promoting health for the patients with T2DM and ESRD in Vietnam.

By supporting patients to take part in their social context, the nurses could promote health for patients with T2DM and/or ESRD. Support from relatives and other patients was described as important when promoting health, and this is consistent with Pender (2011) who emphasizes how expectations and support from others are important to enable a healthy behaviour. A reason for this emphasis on the patients’ social context may be that Vietnam is described as a collectivistic society, where it is important for the individual to know what others think you can do and to adapt to the social norm (Coburn & Weismuller, 2012). Therefore, it should be interesting to furthermore examine patients’ motivators to healthy behaviours, and the efficiency of various health-promoting strategies in the Vietnamese context. Further developing health-promoting strategies, suitable for different contexts, would improve the nurses’ possibilities to promote health in their daily work. The nurses’ health-promoting work for patients with T2DM and/or ESRD needs to be a priority for the health-care settings.

The nurses described barriers in the health-promoting work as a lack of knowledge about health promotion and strategies to promote health for patients with T2DM and/or ESRD. This conception conforms to previous research that has shown that Vietnamese nurses did not consider that their education was complete (Jones et al., 2000; Pron et al., 2008). This indicates that health promotion and health-promoting strategies need to be further integrated in the nursing education as well as the workplace introduction. Furthermore, the nurses need time to practice health promotion in their daily work.

Regarding patients with T2DM and/or ESRD, social context, such as economic conditions, sometimes impeded nurses to promote health. This has also been shown in previous studies, which describe that all patients cannot follow the treatment due to economic conditions (Baumann et al., 2006; Ito et al., 2008), and hospitalization of a family member can lead to an economic disaster for the household (Van Minh, Kim Phuong, Saksena, James, & Xu, 2012). This indicates how further work is necessary when dealing with inequalities in health in Vietnam, but much of this work lies outside the individual nurse's authority.

The nurses described how they could promote health by educating patients with T2DM and/or ESRD as well as their relatives about health, disease, and self-care. Nurses expressed that they promoted health by answering questions, explaining and educating the patients and their relatives. This view is consistent with previous research which describes how nurses view health education and information as the most important intervention to promote health (DeCola, Benton, Peterson, & Matebeni, 2012).

The nurses in the present study described how health education was organized at the hospitals as group education once a week. Therefore, this could be an opportunity to further develop these group meetings to improve education about health, disease, and self-care for patients and their relatives.

Even if the nurses in the present study emphasized the importance of educating the patients with T2DM and/or ESRD about health, disease, and self-care, some nurses described barriers in this work. The nurses described how patients wanted to hear the advice from the doctor; otherwise they did not trust the nurses. This indicates that Vietnamese nurses social status is low. This may partly be explained by the fact that there is no standard exam for nurses and that the level of education varies between different nursing schools and parts of the country (Hill & Crow, 2013). Therefore, it may be advantageous to create a national education program for nurses that also provide knowledge about healthy lifestyles and methods to promote health. The nurses need to be empowered so they are able to work independently to promote the patients’ health. With improved education for nurses’ regarding health promotion and health promotion strategies, it is also possible to make the health promotion work become more efficient.

Furthermore, the conditions at the hospital were also a barrier for the nurses when providing health education and promoting health to the patients with T2DM and/or ESRD. Lack of time, lack of facilities, and overcrowded hospitals were a part of the Vietnamese nurses’ daily work. Lack of time for nurses to promote health has also been shown in other countries, such as Sweden and England (Brobeck, Odencrants, Bergh, & Hildingh, 2013; DeCola et al., 2012). This indicates that there is a need to integrate and raise health promotion in the daily work and allow time for health-promoting nursing, both in Vietnam and in other countries.

The nurses described how they could promote health by supporting patients with T2DM and/or ESRD to be physically active****. Physical activity was emphasized as an important part of a healthy lifestyle, and the nurses thought it was important for the patients as well as the general public. Physical activity was described to improve both physical and mental health and to prevent complications. This view of physical activity, as an important part to promote health, is consistent with descriptions about physical activity being an important issue in the treatment and prevention of T2DM (Eckert, 2012; Ng et al., 2011) and ESRD (Ouzouni et al., 2009; Storer et al., 2005). Ideas such as providing training equipment and sport activities in the hospital area were described by the nurses in the present study, which correlates with ideas about creating a supportive environment and healthy hospitals (Green & Tones, 2010). More resources are needed to actualize many of these ideas in the daily work and to create a supportive environment at the hospitals.

On the contrary, it is described that there is a historical and traditional knowledge about the importance of physical activity in Vietnam; however, this knowledge has been forgotten by the younger generations (Sundberg, 2013). Furthermore, there have been rapid changes in the Vietnamese society which have led to an adoption of a more sedentary lifestyle, where people choose to ride motorbikes instead of the traditional bicycle (Baumann et al., 2006). This indicates that, even if physical activity is considered to be an important intervention to promote health, there are major barriers to conquer for the Vietnamese nurses when advocating for physical activity. The nurses described a lack of space and resources at the hospitals to be barriers to promote physical activity to patients with T2DM and/or ESRD.

This limited the possibilities for the patients to be physically active while they stayed at the hospital. This is in line with Fitzgerald and Spaccarotella's (2009) description of how barriers to physical activity may be linked to socioeconomic characteristics of neighbourhoods and environment. However, it should be obvious that hospitals contribute to the health in the community and provide a healing environment (Hancock, 1999). Furthermore, there is a need for communities to promote physical activity and create supportive environments for active living (Faskunger, 2011). Looking beyond these barriers, there are many opportunities to improve nurses’ health-promoting work, create health-promoting hospitals, and develop a healthy society that supports a physically active living. To further improve nurses’ health-promoting work, health promotion needs to have a more prominent role in the nursing education. There is also a need to strengthen nurses and other people's belief in the nursing knowledge.

## Methodological consideration

For the purpose of ensuring the quality of the data collection and findings, the phenomenographic approach in this study will be discussed in terms of the three criteria of trustworthiness: credibility, transferability, and dependability of findings and methods used (Polit & Beck, 2012).

*Credibility* concerns the sample and the collection method, which should allow the greatest potential for providing maximum information (Polit & Beck, 2012). In order to obtain a varied sample, the present study used a purposeful sample. However, variations in gender were limited, due to the fact that the majority of the nurses at the two units where the study was performed were women. The small amount of men in this study may be explained by the fact that only approximately 8% of Vietnamese nurses are men (Crow & Le, 2011).

The semi-structured interviews were performed with an interview guide, which is in line with Sjöström and Dahlgren's (2002) recommendations. The main difficulties with the present study were the language and the interpretation between English and Vietnamese. According to Foucault (2002), the language is a discourse in itself; a collective code in society which is important for the perception of phenomena. To create a good interpretation of the interviews, the interpreters, who had Vietnamese as their mother tongue and were familiar with the Vietnamese nursing language, followed the development of the interview guide. However, there were difficulties for the author to control the interviews and create a relationship with the informant due to the language barrier; therefore, it was not possible to create in-depth interviews. It was also difficult to create follow-up questions for the same reason. There were also cultural differences and organizational differences between Sweden and Vietnam that sometimes complicated the understanding of the answers. Some follow-up questions were direct questions about the nurses’ conditions in the hospital to understand what their answers referred to. The interview guide could have been improved if more time was disposed for the pilot-interviews, but this was not possible due to the time limits.

Sjöström and Dahlgren (2002) argue that quotations are a way to prove that the categories of descriptions are well-grounded in the data. Therefore, to create credibility for the present study, each conception was strengthened with some quotations from the interviews.

*Dependability* corresponds to reliability in quantitative studies (Polit & Beck, 2012). This was ensured by the fact that the same author conducted all the interviews. In order to achieve a better understanding about nursing and the environment at hospitals in Vietnam, the first author spent some time at the hospitals, but was not involved in the patient care. This was important in order to understand the participants’ answers better. To handle the author's preconceptions during the interviews, all interviews were done with curiosity and many clarifying questions were asked.

The hospitals where the participants worked were considered to be the best place for the interviews. Due to the lack of space and rooms, it was not possible to do the interviews in a separate room. This was perhaps unnecessary; however, other members of staff or patients who spoke to the participants interrupted many of the interviews. This may have caused the participant to lose focus regarding the interview question; however, at the same time, this was part of the environment at the hospital as well as in the Vietnamese society.

*Transferability* handles issues related to how the findings can be transferred to other settings or groups (Polit & Beck, 2012). The transferability of the findings to other healthcare settings in Vietnam is strengthened by the variations in gender, education, and years of nursing experience.

## Conclusion

Nurses described how health was promoted by creating a positive relationship, by supporting patients to take part in their social context, by educating patients and relatives, and by supporting patients to be physically active. Nurses supported patients to connect with relatives and other patients, or take part in patient clubs and campaigns in society. Health education was mainly provided in larger groups with patients and relatives. Barriers to promote health include difficulties in communication with the patients, such as patients with lack of motivation, patients from ethnic minorities creating a language barrier, and patients with mental illnesses. Relatives could both be a resource and a barrier to promote health as well as the patients’ socio-economic status and poverty. Barriers in supporting patients to be physically active include a lack of support devices, training equipment, and space to be physically active.

The findings of this study indicate that there is a need to further integrate knowledge about health promotion in the Vietnamese nursing education. Furthermore, the barriers that nurses face in their daily work indicate that it would be beneficial to develop the nursing education and create a national standard or registration for nurses to improve the social status of the nurses. Further research is necessary to examine Vietnamese patients’, with T2DM and/or ESRD, knowledge and attitudes about health, and the efficiency of various health-promoting strategies in a Vietnamese context.
